# Phylogeny, chromosomal mapping and expression analyses of wheat CLAVATA pathway components suggest differential selection on receptor‐like kinases, CLEs and T3 WOXes

**DOI:** 10.1111/tpj.70580

**Published:** 2025-12-04

**Authors:** Katie Jeal, Sophie J. Carpenter, Chris Burt, Keith J. Edwards, C. Jill Harrison

**Affiliations:** ^1^ School of Biological Sciences University of Bristol 24 Tyndall Avenue Bristol BS8 1TQ UK; ^2^ RAGT Seeds Ltd. Grange Road Ickleton Saffron Walden Essex CB10 1TA UK; ^3^ Present address: Sainsbury Laboratory University of Cambridge 47 Bateman Street Cambridge CB2 1LR UK

**Keywords:** wheat, CLAVATA, phylogenetic analysis, CLE, WOX, CRN, BAM, RPK2

## Abstract

Ensuring continuous global food security is a major challenge of the 21st century. Wheat contributes approximately 20% of the total calories consumed by humans, and an estimated 60% increase in production will be required by 2050 to meet forecast global demand. In cereals like wheat, inflorescence (ear) size and branching patterns determine the number of flowers (florets) and grains produced, and these aspects of plant architecture are regulated by the activity of stem cells in the growing shoot tips. CLAVATA peptide and receptor‐like kinase signalling regulates angiosperm stem cell activity, and as changes in CLAVATA function can improve crop yields, CLAVATA is a key target for reverse engineering. Here, we identify components of the wheat CLAVATA pathway using genome searches against *Triticum aestivum* and its wild relatives *Triticum turgidum* ssp. *durum*, *Triticum turgidum* ssp. *dicoccoides*, *Triticum urartu* and *Aegilops tauschii*. Using phylogenetic and synteny analysis, we determine the relationship between homoeologues and infer patterns of gene family evolution. Whilst *CLAVATA1*, *BARELY ANY MERISTEM*, *RECEPTOR‐LIKE PROTEIN KINASE 2*, *CORYNE* and *CLAVATA2* receptor‐like kinase homologues are mainly present as single genome copies as in other grasses, *CLAVATA3*‐like but not *TRACHEARY ELEMENT DIFFERENTIATION FACTOR* (*TDIF*)‐like peptide encoding genes and *WUSCHEL‐LIKE HOMEOBOX* (*WOX*) genes have expanded copy numbers with many gene gains and losses during evolution. Our results highlight wheat CLAVATA pathway components for reverse genetic analysis and indicate potential differential selection on wheat receptor‐like kinases, their peptide ligands and *WOX*es.

## Significance Statement

The Arabidopsis CLAVATA pathway regulates shoot apex and fruit size, and disrupting CLAVATA function has led to yield improvement in crops. Despite its central position in agriculture, the potential for yield improvement in wheat has not been explored. Translation into wheat requires detailed knowledge of the number and evolutionary history of CLAVATA pathway genes in different cultivars. Here, we identify wheat genes in key families of the wheat CLAVATA pathway.

## INTRODUCTION

**Figure 1:**
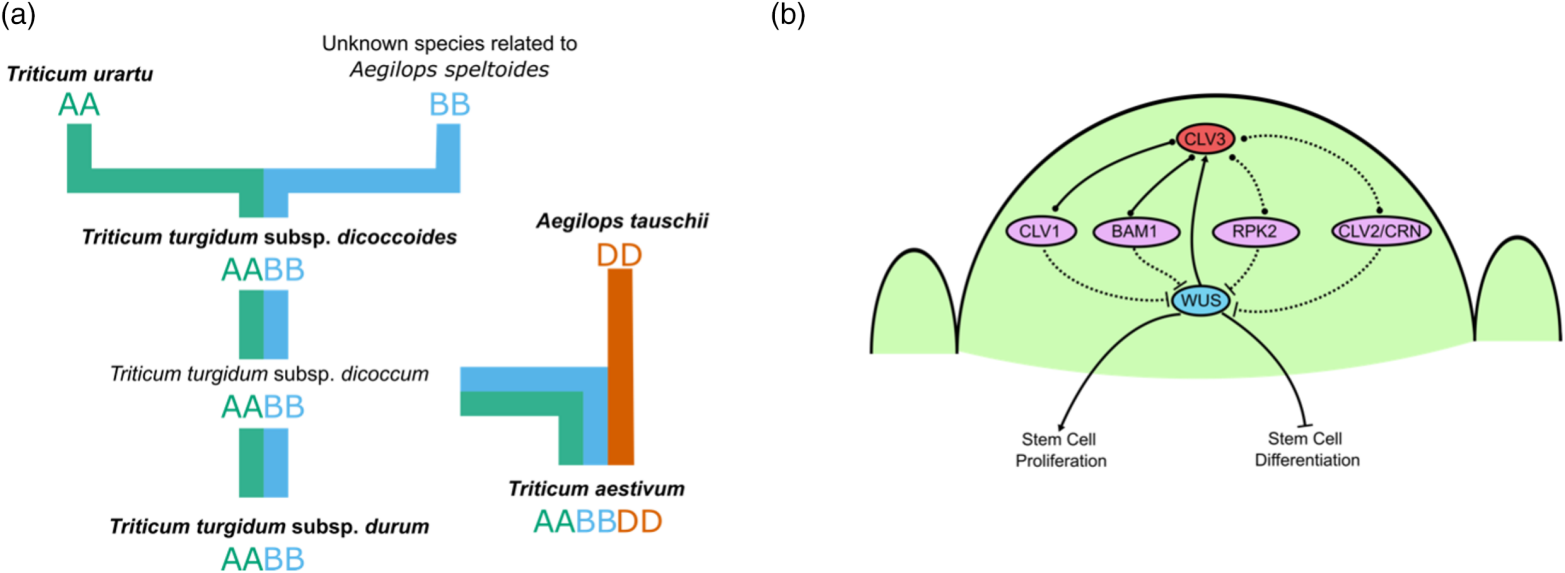
Identifying wheat CLAVATA pathway components. (a) The evolutionary relationships of wheat relatives showing the history of polyploidisation in bread wheat (*Triticum aestivum*) evolution. The *T. aestivum* genome comprises A, B and D genomes derived from *Triticum urartu* (A), an unknown relative of *Aegilops speltoides* (B) and *Aegilops tauschii* (D). Adapted from (Levy & Feldman, 2022). (b) Schematic showing that the canonical *Arabidopsis thaliana* CLAVATA (CLV) pathway comprises CLV3‐like peptides, CLV1, BAM1, RPK2, CLV2 and CRN receptor‐like kinases and the WUSCHEL transcription factor. Solid lines represent direct interactions and dashed lines represent indirect interactions. Arrows represent positive regulation and bars represent negative regulation. Adapted from Fletcher (2018).

Bread wheat (*Triticum aestivum*) is an allopolyploid monocot species of vital importance to the global economy and food security. Its hexaploid nature is the result of multiple hybridisation events between species in the Triticeae tribe (Figure 1a). Approximately 800 000 years ago *Triticum urartu* (red wild einkorn wheat) hybridised with an unidentified relative of *Aegilops speltoides* (a goatgrass), forming the tetraploid *Triticum turgidum* ssp. *dicoccoides* (wild emmer wheat) (Levy & Feldman, 2022). This was cultivated for several thousand years, leading to the establishment of tetraploid wheat subspecies *T. turgidum* ssp. *dicoccum* (domesticated emmer wheat) and *T. turgidum* ssp. *durum* (durum wheat) (Levy & Feldman, 2022). Multiple independent hybridisations between *T. turgidum* ssp. *dicoccum* and *Aegilops tauschii* (Tausch's goatgrass) led to the origin of modern hexaploid bread wheat (*T. aestivum*) 8500–9000 years ago (Levy & Feldman, 2022). *T. aestivum* is now one of the most widely grown crops by area globally (FAOSTAT, 2022), with a 2023 value of £2.9 billion to the UK economy alone (DEFRA, 2024), and durum wheat is also widely cultivated. In the last 10 years, wheat yields have increased by an average of 0.75% per year, plateauing at 788 million tonnes per year (USDA, 2024). However, to feed a projected global human population of 9.7 billion people in 2050 (United Nations, 2019), wheat yield increases of 2–3% per year will be required (Hawkesford et al., 2013). The advancement of scientific knowledge about bread wheat has been hindered by the complexity of wheat genomes and the 4–6 month generation time (Adamski et al., 2020). However, recent advances such as the release of *T. aestivum*, *T. urartu* and *A. tauschii* reference genomes (International Wheat Genome Consortium (IWGSC), 2018; Ling et al., 2013; Luo et al., 2017), and research tools such as the Wheat Expression Browser (Ramírez‐González et al., 2018; Winter et al., 2007), the Wheat Spatial Transcriptomics Browser (Long et al., 2024; Xu et al., 2025), a TILLING population (Krasileva et al., 2017) and CRIPSR Cas9‐mediated gene editing protocols (Wang et al., 2020) unlock significant potential for reverse genetics in yield improvement (Adamski et al., 2020; Borrill, 2020; Fletcher, 2018).

The CLAVATA pathway was first identified in the 1990s as a regulator of *Arabidopsis thaliana* (Arabidopsis) shoot apex and silique (fruit) size (Clark et al., 1996; Laux et al., 1996; Leyser & Furner, 1992), and CLAVATA maintains the size of shoot apices during plant growth through the activity of a molecular feedback loop (Schoof et al., 2000; Somssich et al., 2016) (Figure 1b). In brief, *CLAVATA3* (*CLV3*) is expressed in the central zone of the shoot apex (Fletcher et al., 1999), encoding a protein that is processed to yield a small secreted and diffusible peptide (Kondo et al., 2006; Lenhard & Laux, 2003; Ohyama et al., 2009; Rojo et al., 2002). The peptide acts as a ligand for the CLAVATA1 (CLV1) receptor‐like kinase which is generated in inner layers of the shoot apex (Clark et al., 1997; Ogawa et al., 2008), and *CLV1* has several homologues (*BARELY ANY MERISTEM*; *BAM1*, *BAM2* and *BAM3*) which have some redundancy with each other and with *CLV1* (DeYoung et al., 2006; Nimchuk, 2017; Whitewoods et al., 2018). BAM1 forms further receptor complexes with RECEPTOR‐LIKE PROTEIN KINASE 2 (RPK2) (Kinoshita et al., 2010) and a third receptor complex comprises CLAVATA2 (CLV2) and CORYNE (CRN) (Jeong et al., 1999; Müller et al., 2008; Zhu et al., 2010). CLAVATA3 INSENSITIVE KINASEs (CIKS) act as co‐receptors with all afore mentioned receptor‐like kinases to mediate phosphorylation in CLV3 signalling (Hu et al., 2018). Further signalling components acting downstream of CLV1 include the POLTERGEIST (POL) and POL‐like protein phosphatases (Gagne & Clark, 2007; Song et al., 2006), the PBL34/35/36 cytoplasmic kinases (DeFalco et al., 2022; Wang et al., 2022) and the MAZZA cytoplasmic kinase (Blümke et al., 2021). Such CLV1 signalling results in suppression of expression of the *WUSCHEL* (*WUS*) gene at the centre of the shoot apex (Schoof et al., 2000). The WUSCHEL transcription factor moves via plasmodesmata to the surface layers of the shoot apex (Daum et al., 2014; Yadav et al., 2011) where it promotes the expression of *CLV3* (Brand et al., 2002). As *clv* mutants have an enlarged apical stem cell pool and *wus* mutant shoot apices terminate prematurely (Clark et al., 1997; Laux et al., 1996), fine‐tuning of the *CLV*/*WUS* feedback loop can lead to changes in the size of the shoot apex, and consequent changes in organ initiation without gross perturbations in overall plant form (Schoof et al., 2000).

Broader potential for intercepting CLAVATA function by reverse genetics to improve crop yields was recently highlighted in ‘The CLV‐WUS stem cell signalling pathway: a roadmap to crop yield optimisation’ (Fletcher, 2018). In *Brassicas*, where seeds are pressed for oil, the size of the silique determines the number of seeds and hence yield (Diepenbrock, 2000). Whilst there are normally two locules per silique, loss of function *CLAVATA* mutations can lead to an increased number of locules and higher yields without detriment to overall viability (Fan et al., 2014; Xiao et al., 2018; Xu et al., 2017). Similarly, in tomato, downregulation of CLAVATA function can lead to an increase in fruit size, locule number and yield (Chu et al., 2019; Xu et al., 2015), and *CLAVATA* mutations underwent selection during tomato domestication (Lippman & Tanksley, 2001; Muños et al., 2011). In grasses, yield variability in mutants with disrupted CLV/WUS function reflects changes in inflorescence architecture rather than fruit size (Bommert & Whipple, 2018; Fletcher, 2018; McKim et al., 2018; Zhang & Yuan, 2014). In maize, where each kernel arises from a floral meristem on the female inflorescence (the ear), *CLAVATA* (*FASCIATED EAR2*; *FEA2*, *FEA3* and *THICK TASSEL DWARF*; *TD1*) and *WUSCHEL* mutations affect the size of the ear meristem, and hence the number of subsequently developing spikelet pair, spikelet meristems, floral meristems and kernels (Bommert et al., 2005; Chen et al., 2021; Je et al., 2016, 2018; Taguchi‐Shiobara et al., 2001), and CLAVATA pathway components were likely targets of selection during domestication (Doebley et al., 2006). The rice inflorescence is a panicle with primary branches formed by panicle branch meristems and secondary branches formed by spikelet branch meristems; both can initiate spikelet meristems which differentiate into floret meristems (McKim et al., 2018). Rice *CLV1* (*FLORAL ORGAN NUMBER 1; FON1*) and *CLV3* (*FLORAL ORGAN NUMBER 2/4; FON2/FON4*)orthologues affect inflorescence meristem size and architecture, floret meristem size and floral organ number (Chu et al., 2006; McKim et al., 2018; Suzaki et al., 2004), whilst the *WUS* orthologue (*TILLERS ABSENT*) promotes stem cell fate in axillary meristems and is required for ovule development (Tanaka et al., 2015, 2021; Tanaka & Hirano, 2020). The barley inflorescence is a spike which laterally initiates triple spikelet meristems. These form central and lateral spikelet meristems which develop into floral meristems (McKim et al., 2018). The barley *CLV1* orthologue (*HvCLV1*) similarly affects meristem size, and *Hvclv1* mutants have supernumerary spikelets and florets (Vardanega et al., 2025). As in barley, the wheat inflorescence is a spike. The inflorescence meristem gives rise to spikelet meristems, which in turn initiate lateral floral meristems differentiating into florets (McKim et al., 2018), and roles for CLAVATA are not yet known.

Here we used BLAST (Altschul et al., 1990), Ensembl Plants Compara (Harrison et al., 2023) and OrthoFinder2 (Emms & Kelly, 2019) to search genome databases of *T. aestivum*, its wild relatives *T. turgidum* ssp. *durum*, *T. turgidum* ssp. *dicoccoides*, *T. urartu* and *A. tauschii*, and rice (*Oryza sativa*), maize (*Zea mays*) and Arabidopsis as outgroups. We identified 3 *CLV1*, 15 *BAM*, 3 *CLV2*, 3 *CRN*, 9 *RPK2*, 112 *CLAVATA3/ENDOSPERM SURROUNDING REGION*‐related (*CLE*) and 26 T3 *WOX* genes (homologues of Arabidopsis *WOX*es 1–7 and *WUS*) in *T. aestivum* and explored the evolutionary history of each of these genes using phylogenetic analysis and synteny analysis. We also used eFP Browser data to examine expression level differences between A, B and D genome copies of each gene (Ramírez‐González et al., 2018; Winter et al., 2007). For the wheat receptor‐like kinases, A, B and D genome copies of each gene were mostly resolved as sisters to progenitor genomes and retained at grass ancestral baseline copy numbers, suggesting a lack of directional selection or that selection is stabilising. However, *CLE* and T3 *WOX* copy numbers were more variable, showing recent expansions and losses in wheat, relationships between different genome copies were also more variable and *TaCLE* expression levels were less balanced between genome copies than other genes. Our work identifies genes for reverse genetic investigations of CLAVATA function and suggests potential for differential selection on wheat receptor‐like kinases, *CLE*s and T3 *WOX*es.

## RESULTS

### BLAST and phylogenetic analysis identified three *TaCLV1* genes, nine *TaBAM1/2* genes and six *TaBAM3* genes

**Figure 2:**
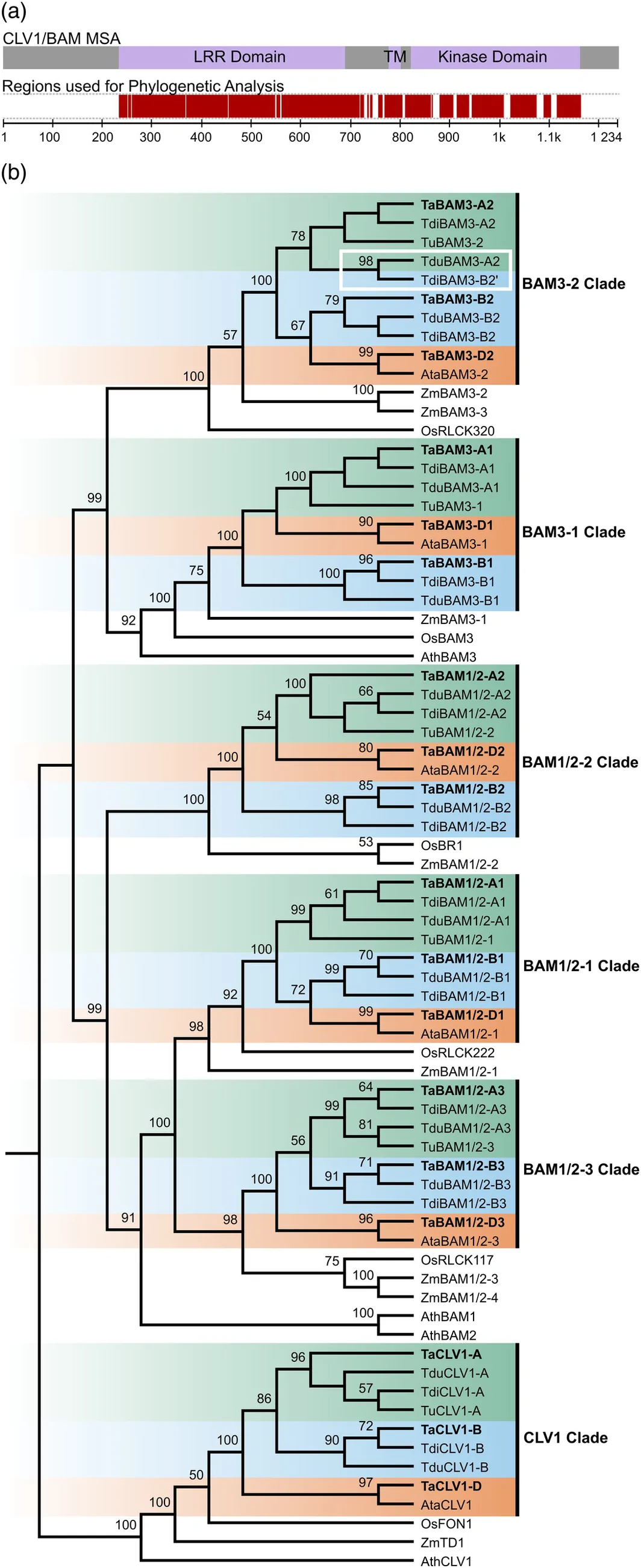
Phylogenetic relationships between Triticeae CLV1 and BAM1/2/3 homologues. (a) Schematic representing the structure of 73 CLV1 and BAM‐like amino acid sequences that were aligned using MUSCLE (Edgar, 2004), with domains annotated against the Arabidopsis sequence. Regions in red represent conserved sequences used for maximum likelihood tree generation. LRR, leucine rich repeat; MSA, multiple sequence alignment; TM, transmembrane. Scale bar shows amino acid position. (b) A maximum likelihood tree with 1000 bootstrap replicates was generated using MEGA11. The resultant tree was midpoint rooted and shows bootstrap values >50; *Arabidopsis thaliana*, *Zea mays* and *Oryza sativa* sequences were included for outgroup comparison. Triticeae genes from *Triticum urartu* and *Triticum aestivum* A subgenomes are highlighted in green, genes from the *Triticum turgidum* and *T. aestivum B* subgenomes are highlighted in blue and genes from *Aegilops tauschii* and *T. aestivum* D genomes are highlighted in orange. *T. aestivum* genes are indicated in bold. The white box indicates the position of a *Triticum durum* BAM sequence that did not form a clade with other B genome sequences. Genes were named following wheat guidelines (Boden et al., 2023) and in relation to Arabidopsis outgroups.

In Arabidopsis, CLV1 and its closely related proteins BAM1, BAM2 and BAM3 act with some redundancy in the SAM to perceive CLV3 and repress *WUS* expression (DeYoung et al., 2006; Deyoung & Clark, 2008; Lenhard & Laux, 2003; Rodriguez‐Leal et al., 2019; Schlegel et al., 2021; Shinohara & Matsubayashi, 2015). To identify wheat *CLV1* and *BAM* homologues, tBLASTn searches (Altschul et al., 1990) were conducted using Arabidopsis CLV1 and BAM1, BAM2 and BAM3 peptide sequences as queries to search *T. urartu*, *T. turgidum* ssp. *dicoccoides*, *T. turgidum* ssp. *durum*, *T. aestivum* and *A. tauschii* genomes (Tables S1 and S2). Hits with a cut‐off value of e^−1000^ from the initial tBLASTn searches were validated by reciprocal BLAST (Altschul et al., 1990), OrthoFinder2 (Emms & Kelly, 2019) searches and analyses with the Ensembl Plants Compara tool (Harrison et al., 2023) (see Methods section), and hits retrieving the gene of interest from at least two of these methods were taken forward for phylogenetic analysis. Searches against *O. sativa* and *Z. mays* genomes were also performed to retrieve sequences for outgroup comparison, and a total of 55 Triticeae CLAVATA1/BAM‐like sequences were identified (Table S2). To determine relationships between wheat CLAVATA1/BAM homologues and genes with well characterised functions in other species, a full‐length protein sequence alignment was generated using MUSCLE (Edgar, 2004), and the full alignment comprised 1234 positions and positions where more than 10% of sequences contained gaps were removed. This left 775 variable and 34 invariant positions in the final alignment. Conserved regions including the leucine‐rich repeat, transmembrane and kinase domains were used for maximum likelihood tree generation (Figure 2a; Dataset 1) and the resulting tree was midpoint rooted (Figure 2b).

This analysis resolved Triticeae CLV1, BAM1/2 and BAM3 clades that were nested within rice and maize or rice, maize and Arabidopsis sisters (Figure 2b). Two further Triticeae clades (BAM1/2‐2 and BAM3‐2) were nested within monocot clades and were sister to the clades containing Arabidopsis BAM1/2 and BAM3 proteins respectively. Newly identified Triticeae genes therein were named according to their homology to CLV1, BAM1/2 or BAM3. For most Triticeae, the number of genes identified corresponded to species' ploidy levels. For example, a single *CLV1* homologue was found in the diploid *A. tauschii* genome, while two *CLV1* homologues were found in the tetraploid *T. turgidum* ssp. *durum* genome and three *CLV1* homologues were found in the hexaploid *T. aestivum* genome. Excepting a single gene in the *BAM3‐2* clade (white box in Figure 2b), phylogenetic analyses showed that genes from each subgenome and subgenome donor species were more closely related to each other than to genes from other subgenomes or donor species. Thus, all Triticeae A genome *CLV1* genes formed a sister clade to B genome genes, and these sister A and B clades were jointly sister to a D genome clade. This pattern of sister relationship was also shown in the BAM1/2‐3 clade (Figure 2b). However, in BAM1/2‐2 and BAM 3‐1 clades, A and D genome sequences formed sister clades that were jointly sister to B genome sequences, and in the BAM1/2‐1 clade, B and D genome sequences formed sister clades that were jointly sister to an A clade (Figure 2b). An additional *BAM3‐2* gene was identified on the B genome of *T. turgidum* ssp. *durum*, suggesting a possible gene duplication within the *T. turgidum* ssp. *durum* genome (Figure 2b).

### BLAST and phylogenetic analysis identified three *TaCLV2* genes and three *TaCRN* genes

**Figure 3:**
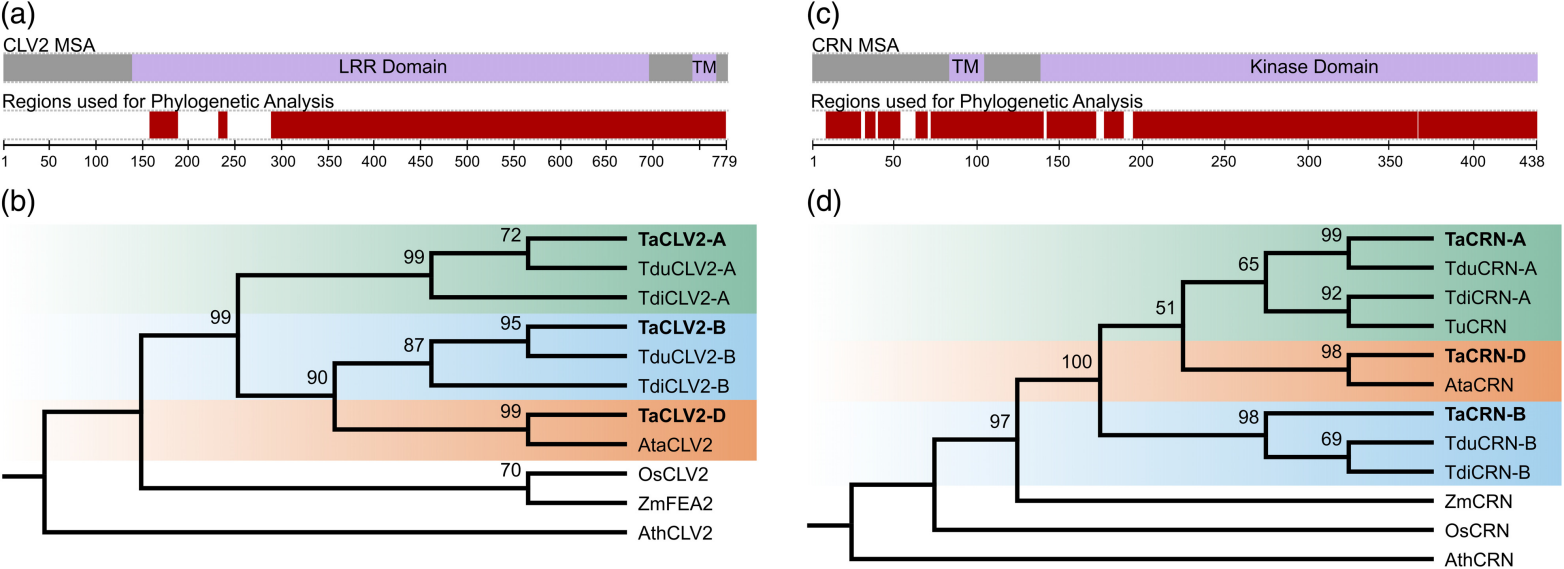
Phylogenetic relationships between Triticeae CLV2 and CRN homologues. (a) Schematic representing the structure of 11 CLV2‐like amino acid sequences. Domains are annotated by comparison to Arabidopsis sequences. LRR, leucine rich repeat; MSA, multiple sequence alignment; TM, transmembrane. Scale bar shows amino acid position. Regions in red represent the conserved regions used for maximum likelihood tree generation. (b) A maximum likelihood CLV2 tree with 1000 bootstrap replicates generated using MEGA11, rooted on Arabidopsis CLV2. Bootstrap values >50 are shown. (c) Schematic representing the structure of 12 CRN‐like coding amino acid sequences. Abbreviations as in (a), and regions in red represent the conserved regions used for maximum likelihood tree generation. (d) A maximum likelihood CRN tree with 1000 bootstrap replicates generated using MEGA11, rooted on Arabidopsis CRN. Bootstrap values >50 are shown. *Arabidopsis thaliana*, *Zea mays* and *Oryza sativa* sequences were included for outgroup comparison. Triticeae genes from *Triticum urartu* and *Triticum aestivum* A subgenomes are highlighted in green, genes from the *Triticum turgidum* and *T. aestivum B* subgenomes are highlighted in blue and genes from *Aegilops tauschii* and *T. aestivum* D genomes are highlighted in orange. *T. aestivum* genes are indicated in bold. Genes were named following wheat guidelines (Boden et al., 2023) and in relation to Arabidopsis outgroups.

In Arabidopsis, CLV2 and CRN jointly act in a co‐receptor complex with CLV1 to repress *WUS* expression (Müller et al., 2008; Shinohara & Matsubayashi, 2015). To identify wheat *CLV2* and *CRN* gene homologues, tBLASTn searches (Altschul et al., 1990) were conducted using Arabidopsis CLV2 and CRN peptide sequences as queries to search *T. urartu*, *T. turgidum* ssp. *dicoccoides*, *T. turgidum* ssp. *durum*, *T. aestivum* and *A. tauschii* genomes (Tables S3 and S4). Hits with a cut‐off value of e^−1000^ from initial tBLASTn searches were validated by reciprocal BLAST (Altschul et al., 1990), OrthoFinder2 (Emms & Kelly, 2019) searches and analyses with the Ensembl Plants Compara tool (Harrison et al., 2023) (see Methods section), and hits retrieving the gene of interest from at least two of these methods were taken forward for phylogenetic analysis. Searches against *O. sativa* and *Z. mays* genomes were also performed to retrieve sequences for outgroup comparison. A total of 11 CLV2‐like and 12 CRN‐like sequences were retrieved, and 8 Triticeae *CLV2* and 9 Triticeae *CRN* genes were identified (Tables S3 and S4). Sequences were aligned using MUSCLE (Edgar, 2004) as described for CLAVATA1, and a full‐length CLAVATA2 protein sequence alignment comprising 779 positions was edited to remove gaps that were present in more than 20% of sequences, leaving 318 variable and 203 invariant positions for the final alignment. Conserved regions including the leucine‐rich repeat and transmembrane domain were used for maximum likelihood tree generation (Figure 3a; Dataset 2) and the resulting tree was rooted on the Arabidopsis CLV2 sequence (Figure 3b). The CRN protein sequence alignment, comprising 438 positions was edited to remove gaps that were present in more than 20% of sequences, leaving 252 variable and 151 invariant positions for the final alignment. Conserved regions including the transmembrane and kinase domains were used for maximum likelihood tree generation (Figure 3c; Dataset 3) and the resulting tree was rooted on the Arabidopsis CRN sequence (Figure 3d). The analyses resolved Triticeae CLV2 (Figure 3c) and CRN (Figure 3d) clades that were nested within rice, maize and Arabidopsis sisters and genes were named following wheat guidelines (Boden et al., 2023) and in relation to Arabidopsis outgroups. For most Triticeae, the number of genes identified corresponded to each species' ploidy level; however, no *CLV2* orthologue was identified in *T. urartu*, suggesting a likely gene loss post‐dating hybridisation events. The topology of the CLV2 tree shows CLV2 B genome sequences forming a sister clade to D genome sequences and jointly sister to the A genome clade. In contrast, the CRN tree shows A and D genome sequences forming a sister clade which is jointly sister to the B genome clade.

### BLAST and phylogenetic analysis identified nine *TaRPK2* genes

**Figure 4:**
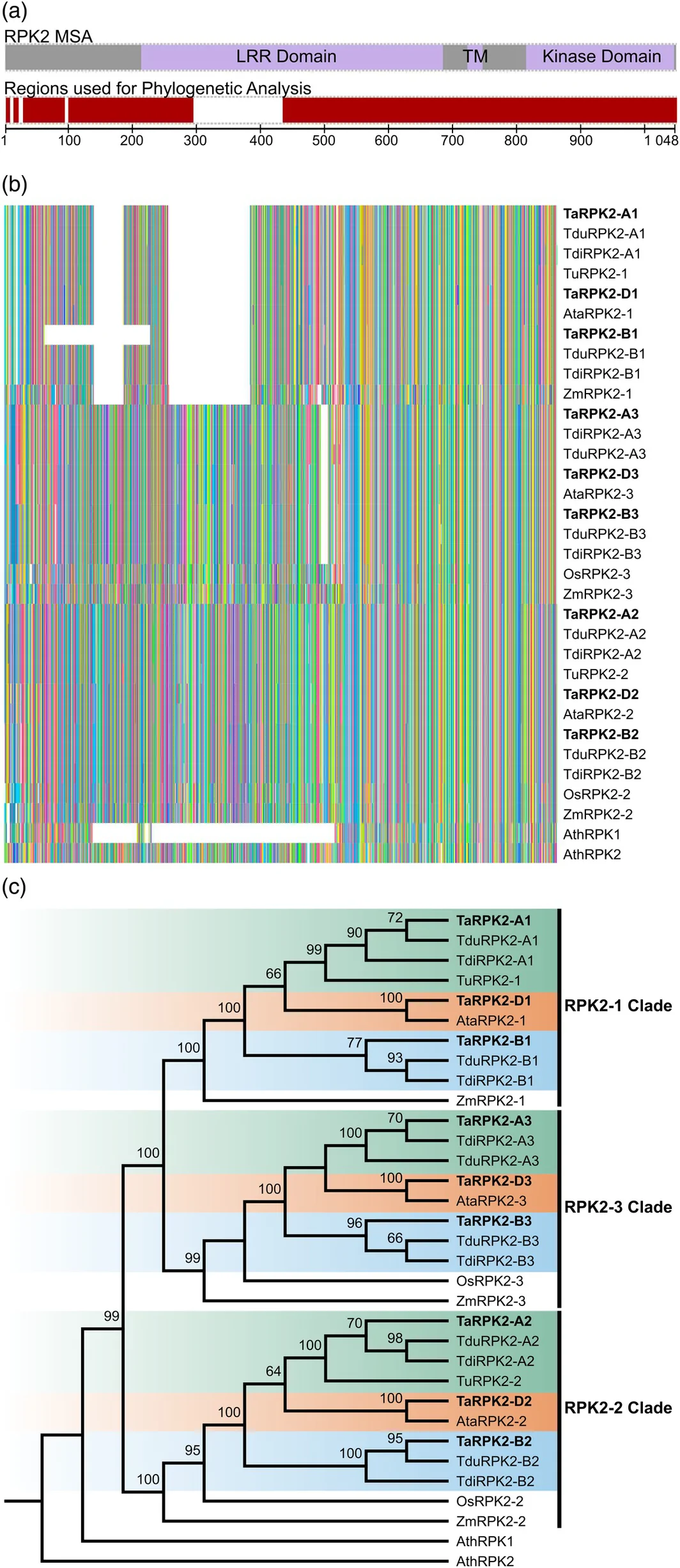
Phylogenetic relationships between Triticeae RPK2 homologues. (a) Schematic representing the structure of RPK2 and the position of conserved aligned amino acid sequences used in phylogenetic analysis. LRR, leucine rich repeat; MSA, multiple sequence alignment; TM, transmembrane. Scale bar shows amino acid position. (b) Screenshot of the multiple sequence alignment used in tree generation showing positions of truncations in the Arabidopsis RPK1 protein and Triticeae RPK2 proteins. (c) A maximum likelihood tree was generated using MEGA11 with 1000 bootstrap replicates and rooted on Arabidopsis RPK2. Bootstrap values >50 are shown. *Arabidopsis thaliana*, *Zea mays* and *Oryza sativa* sequences were included for outgroup comparison. Triticeae genes from *Triticum urartu* and *Triticum aestivum* A subgenomes are highlighted in green, genes from the *Triticum turgidum* and *T. aestivum B* subgenomes are highlighted in blue and genes from *Aegilops tauschii* and *T. aestivum* D genomes are highlighted in orange. *T. aestivum* genes are indicated in bold. Genes were named following wheat guidelines (Boden et al., 2023) and in relation to Arabidopsis RPK2.

In Arabidopsis, RPK2 and BAM1 form a third receptor complex, perceiving CLV3 and triggering downstream signalling that suppresses *WUS* expression (Kinoshita et al., 2010; Shimizu et al., 2015). To identify wheat *RPK2* homologues, tBLASTn searches (Altschul et al., 1990) were conducted using Arabidopsis RPK2 peptide sequences as queries against the species of interest and validated as described for CLV1, CLV2 and CRN (Table S2). Hits with a cut‐off value of e^−1000^ from initial tBLASTn searches were validated by reciprocal BLAST (Altschul et al., 1990), OrthoFinder2 (Emms & Kelly, 2019) searches and analyses with the Ensembl Plants Compara tool (Harrison et al., 2023), and hits retrieving the gene of interest from at least two of these methods were taken forward for phylogenetic analysis (Figure 4). A total of 26 Triticeae RPK2‐like sequences were identified (Table S5) and 7 RPK2‐like sequences were retrieved for rice (2), maize (3) and Arabidopsis (2). These included RPK1, an Arabidopsis RPK2 orthologue which has a truncated LRR kinase domain and acts with RPK2 in embryonic patterning (Cammarata, 2021; Nodine & Tax, 2008).

To determine relationships between wheat RPK2 homologues and genes with well‐characterized functions in other species, a full‐length protein sequence alignment was generated using MUSCLE (Edgar, 2004). Positions comprising more than 35% gaps were excluded, resulting in a final alignment comprising 897 positions, 165 invariant positions and 732 variable positions (Figure 4a,b; Dataset 4). A higher gap threshold was chosen than for other trees to enable inclusion of truncated LRR domains that were phylogenetically informative. Conserved regions including the leucine‐rich repeat, transmembrane and kinase domains were used in maximum likelihood analysis (Figure 4b) and the resulting tree was outgroup rooted on Arabidopsis RPK2 (Figure 4c). Phylogenetic analysis resolved three Triticeae RPK2 clades that were nested within maize or maize and rice sisters (Figure 4c). Similarly to Arabidopsis RPK1, the Triticeae RPK2‐1 clade comprised truncated sequences lacking around 55% of the LRR domain (Figure 4b). However, this clade was nested within non‐truncated Triticeae RPK2‐2 and RPK2‐3 clades, which suggests independent loss of LRR sequence to the Arabidopsis *RPK1* gene. Therefore, all Triticeae genes were named by their homology to RPK2 following wheat nomenclatural guidelines (Boden et al., 2023). In all Triticeae RPK2 clades, A and D genome homoeologues formed sister clades that were jointly sister to the B homoeologues. No *RPK2‐3* gene was identified in *T. urartu*, but for all other Triticeae, the number of genes identified in each species corresponded to ploidy levels.

### BLAST and phylogenetic analysis identified 112 *TaCLE* genes

**Figure 5:**
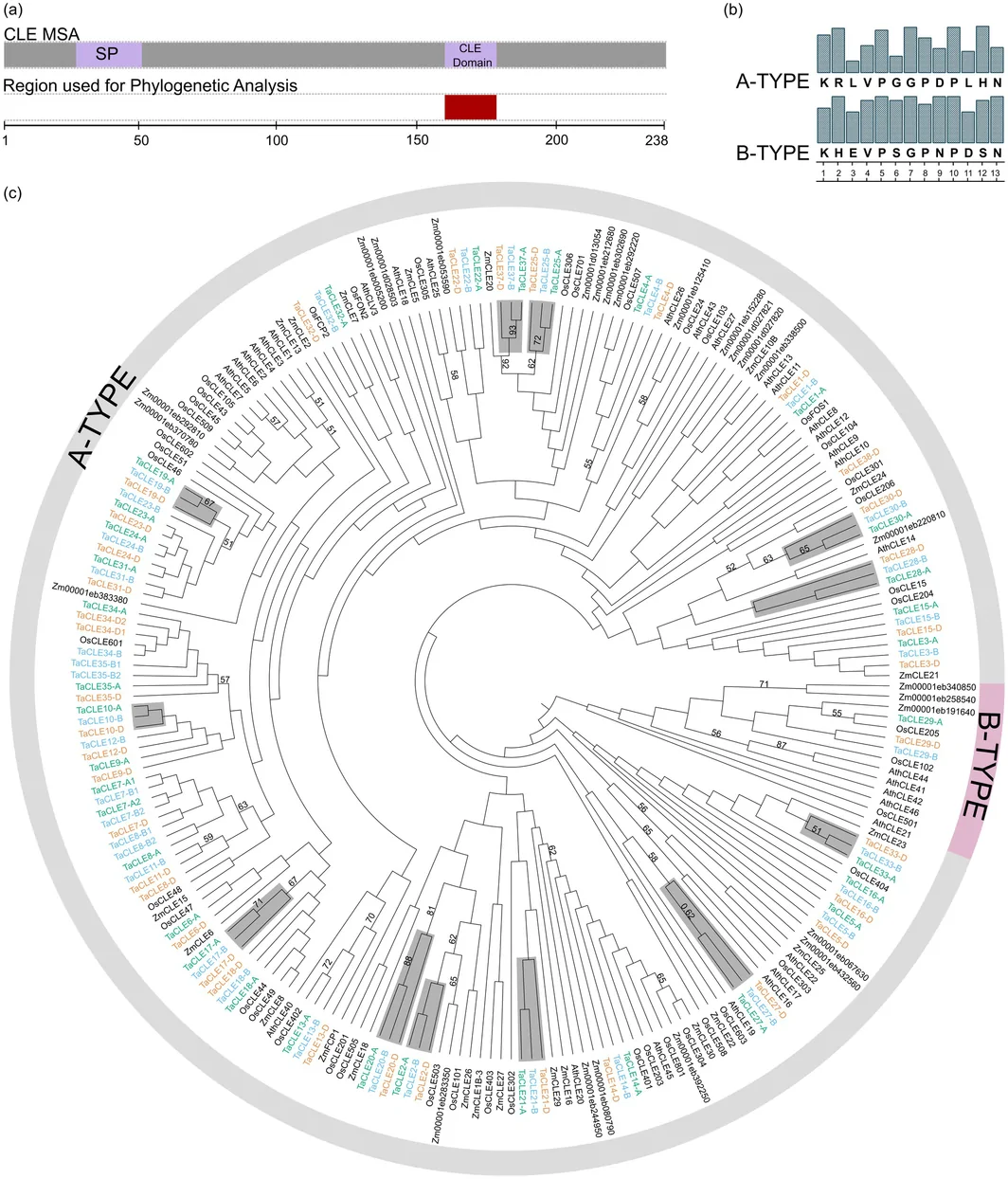
Phylogenetic relationships between *Triticum aestivum* CLE homologues. (a) Schematic representing *CLE* structure with domains annotated against the Arabidopsis *CLV3* sequence, and the position of amino acid sequences used for phylogenetic analysis shown in red. MSA, multiple sequence alignment; SP, signal peptide. Scale bar shows amino acid position. (b) Consensus sequences for A‐type and B‐type CLE domains in our alignments. Scale bar represents amino acid positions. (c) A CLE domain neighbour joining tree with 1000 bootstrap replicates was generated using TreeViewer (Bianchini & Sánchez‐Baracaldo, 2024). The resultant tree was rooted on the B‐type CLE clade and shows bootstrap values >50; *Arabidopsis thaliana*, *Zea mays* and *Oryza sativa* sequences were included for outgroup comparison. *T. aestivum* sequences from the A subgenome are highlighted in green text, sequences from the B subgenome are highlighted in blue and sequences from the D subgenome are highlighted in orange. A, B and D genome triplet clades are highlighted in dark grey. Previously identified *T. aestivum* genes were named following precedent (Li et al., 2019) and newly identified genes were named for their homoeology to named genes or following wheat nomenclature guidelines (Boden et al., 2023). The A‐type CLEs are indicated in grey and B‐type CLEs are indicated in pink.

Whilst there are no previously published wheat CLV1, CRN or RPK2 phylogenies, a previously published paper identified 104 *TaCLE*s and demonstrated their function in stem and root development (Li et al., 2019). However, this paper used different sampling and search strategies from our work. To identify wheat *CLE* homoeologues we therefore conducted tBLASTn searches (Altschul et al., 1990) using Arabidopsis CLE peptide sequences as queries against the species of interest. Hits with a cut‐off value of e^−100^ were validated using methods previously described. A total of 281 Triticeae *CLE*‐like sequences were identified, with 112 in *T. aestivum*, 50 in *T. turgidum* ssp. *durum*, 53 in *T. turgidum* ssp. *dicoccoides*, 30 in *T. urartu* and 36 in *A. tauschii*. Eight previously unknown *TaCLE* genes were identified, *TaCLE33‐A*, *TaCLE34‐B*, *TaCLE36‐D1*, *TaCLE36‐D2*, *TaCLE37‐A*, *TaCLE37‐B*, *TaCLE37‐D* and *TaCLE38‐D*, and all genes retrieved were named following their homoeology to previously named *TaCLEs* (Li et al., 2019), or following wheat nomenclature guidelines (Boden et al., 2023). A total of, 47 rice and 48 maize CLE sequences as reported by Goad et al. (2017) were also included. Due to the large number of genes retrieved from all Triticeae species and limited sequence variation within the CLE motif, only *T. aestivum* sequences were taken forward for tree reconstruction (Tables S6 and S7). All *T. aestivum* sequences contained a CLE domain, but some CLE‐like sequences in wheat, rice and maize contained multiple CLE domains, so these genes (*TaCLE26‐A*, *TaCLE26‐B*, *TaCLE26‐D*, *TaCLE36‐D1*, *TaCLE36‐D2*, *OsCLE502*, *OsCLE504*, *OsCLE506* and *ZmCLE14*) were excluded from analysis. As CLEs have limited sequence conservation outside the 13 amino acid long conserved CLE domain, phylogenetic analysis using methods such as maximum likelihood is inappropriate, and phenetic methods such as CLANS clustering (Goad et al., 2017) or neighbour joining (Whitewoods et al., 2018) have previously been used. We explored both clustering strategies using *T. aestivum*, Arabidopsis, maize and rice protein sequences, and obtained broadly congruent results with each. For ease of visualisation of gene names and groups, we include a neighbour joining tree generated in TreeViewer and rooted on the B‐Type CLEs here (Bianchini & Sánchez‐Baracaldo, 2024) (Figure 5a; Dataset 5). Although support was low or lacking throughout the tree, the B‐Type CLEs formed a distinct clade to A‐Type CLEs, having respective KHEVPSGPNPDSN and KRLVPGGPDPLHN consensus sequences (Figure 5b). The B‐type clade and consisted of the characterised B‐type Arabidopsis, maize and rice genes and the *T. aestivum* gene, *TaCLE29* (Li et al., 2019). The A‐type clade consisted of all other identified *TaCLE*s (Figure 5c), and 12 triplet clades were formed: *TaCLE2*, *TaCLE10*, *TaCLE17*, *TaCLE19*, *TaCLE20*, *TaCLE21*, *TaCLE25*, *TaCLE27*, *TaCLE28*, *TaCLE30*, *TaCLE33* and *TaCLE37*. In all triplet clades, A and B genome copies were sister to each other and jointly sister to the D copy. All other wheat genes were paraphyletic to genes from other species (e.g. *TaCLE3*, *TaCLE14*) or formed clades with multiple genes from different wheat genomes (e.g. *TaCLE7*, *TaCLE8*, *TaCLE11*).

### BLAST and phylogenetic analysis identified eight TaWOX clades in the T3 WOX family

**Figure 6:**
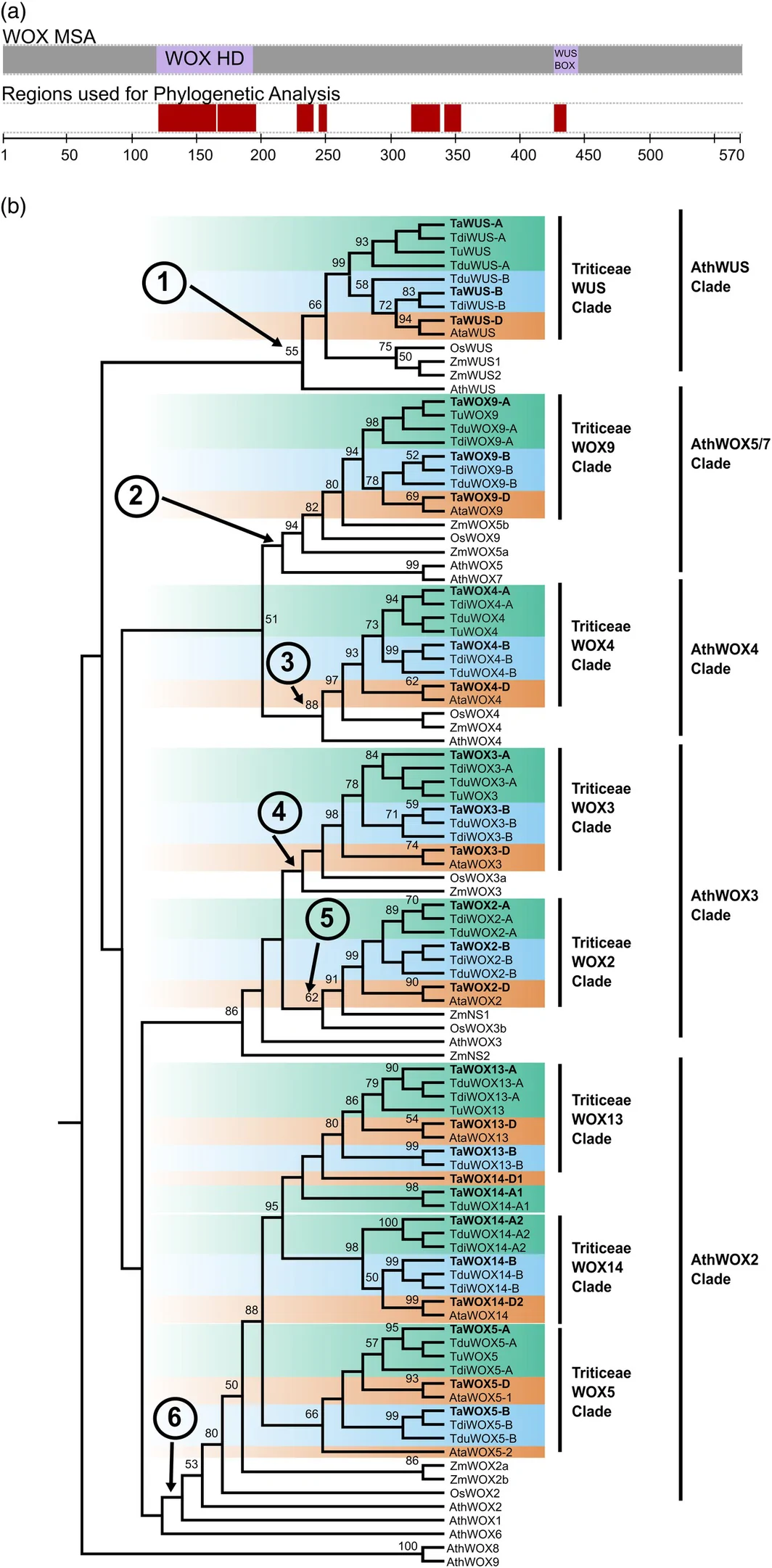
Phylogenetic relationships between Triticeae T3 WOX homologues. (a) Schematic representing WOX structure with domains annotated against the Arabidopsis sequence, and the position of amino acid sequences used for phylogenetic analysis shown in red. MSA, multiple sequence alignment; WOX HD, WOX homeodomain; WUS BOX, WUS Box Motif (van der Graaff et al., 2009). Scale bar shows amino acid position. (b) A maximum likelihood tree with 1000 bootstrap replicates was generated using MEGA11, identifying five clades with Arabidopsis sister WOXes. The resultant tree was outgroup rooted on the AthWOX8 and AthWOX9 clade and shows bootstrap values >50; *Arabidopsis thaliana*, *Zea mays* and *Oryza sativa* sequences were included for outgroup comparison. Triticeae genes from *Triticum urartu* and *Triticum aestivum* A subgenomes are highlighted in green, genes from the *Triticum turgidum* and *T. aestivum B* subgenomes are highlighted in blue and genes from *Aegilops tauschii* and *T. aestivum* D genomes are highlighted in orange. *T. aestivum* genes are indicated in bold. *T. aestivum* genes were named following precedent (Li et al., 2020; Shi et al., 2021) and newly identified Triticeae genes were named in relation to the *T. aestivum* genes.

In Arabidopsis, *WUSCHEL* acts downstream of CLAVATA signalling to promote stem cell identity (Schoof et al., 2000), and *WUSCHEL* is part of a larger *WUSCHEL*‐related homeobox (*WOX*) family comprising 14 members (van der Graaff et al., 2009). Phylogenomic analysis revealed that *WOX* genes form T1, T2 and T3 superclades, and that *WUSCHEL* is a member of the euphyllophyte‐specific T3 *WOX* clade (Wu et al., 2019). To identify wheat T3 *WOX* homologues, tBLASTn searches (Altschul et al., 1990) were conducted using Arabidopsis T3 WOX peptide sequences (AthWUS and AthWOXes 1–7) as queries against the species of interest. Hits with a cut‐off value of e^−100^ from initial tBLASTn searches (Altschul et al., 1990) were validated as previously described. A total of 73 Triticeae T3 WOX‐like sequences were identified, with 26 in *T. aestivum*, 17 in *T. turgidum* ssp. *durum*, 15 in *T. turgidum* ssp. *dicoccoides*, 6 in *T. urartu* and 9 in *A. tauschii*. A total of, 6 rice and 10 maize T3 WOX sequences were also retrieved (SI Table 8). The highly conserved eight amino acid WUS‐box motif that typifies T3 WOX family members (Haecker et al., 2004; van der Graaff et al., 2009; Wu et al., 2019) was found in all Triticeae sequences except AtaWOX5‐2, TiWOX9‐A, TdiWOX5‐A and TuWOX3. To determine relationships between Triticeae T3 WOX homologues and proteins with well characterised functions in other species, a full‐length protein sequence alignment was generated initially using MUSCLE (Edgar, 2004). As this did not align the conserved WUS‐box well, alignments were manually edited using UniproUGENE (Okonechnikov et al., 2012). Positions where more than 10% of sequences contained gaps were removed, leaving 109 variable and 7 invariant positions in the final alignment (Dataset 6). Conserved regions including the WOX homeodomain and WUS‐box motif were used for maximum likelihood tree generation (Figure 6a; Dataset 6) and the resulting tree was rooted on Arabidopsis WOX8 and WOX9 sequences as T2 WOX outgroups (Figure 6b). Wheat sequences were named according to previously published work identifying TaWOX proteins (Li et al., 2020; Shi et al., 2021) and genes from other Triticeae species were named according to their relationship to TaWOXes.

Phylogenetic analysis resolved 6 Triticeae T3 WOX clades that were nested within Arabidopsis, maize and rice or maize and rice sisters (Figure 6b). An AthWUS clade (Triticeae WUS) was sister to all other T3 WOX clades. Two monocot AthWOX3‐like clades including Triticeae sequences (Triticeae WOX2 and Triticeae WOX3) were sisters (Figure 6b). In a clade of AthWOX2‐like sequences, Triticeae WOX13 and WOX14 clades were sisters, and were jointly sister to a WOX5 clade. The AthWOX2 clade was sister to the AthWOX3 clade, and these clades were jointly sister to AthWOX5/7 and AthWOX4 clades. Triticeae WOX2, WOX3 and WOX 4 clades had A and B genome subclades as sisters and joint sisters to a D genome subclade. In the Triticeae WOX2 and WOX3 clades, Triticeae sequences were nested within maize and rice homologues, suggesting that there was a gene duplication in grasses (Figure 6b). No *T. urartu* Triticeae *WOX2* gene was identified. The Triticeae WUS, WOX9 and WOX14 clades resolved B and D genome sequences as sisters and joint sister clades to A subclades (Figure 6b), and the Triticeae WOX5 and WOX13 clades resolved A and D subclades as sisters and joint sisters to B genome subclades (Figure 6b). The Triticeae WOX5 clade was sister to a further wheat sequence, AtaWOX5‐2, which lacks a WOX motif. As this sequence represents an increase in copy number in *A. tauschii*, it likely arose from a recent duplication in that species, and speculatively, it may be pseudogenising. The Triticeae WOX13 clade also had further wheat sisters, TaWOX14‐D1, TaWOX14‐A1 and TduWOX14‐D1. We followed nomenclatural precedent (Li et al., 2020) rather than phylogenetic relationships in naming these genes. No Triticeae WOX13 was identified on the B genome of *T. turgidum* ssp. *dicoccoides* and no Triticeae WOX14 orthologous to TaWOX14‐A1 and TduWOX14‐A1 was identified on the A genome. No *T. urartu* WOX14 was identified. As in previously published work (Wu et al., 2019), no Triticeae orthologues of Arabidopsis WOX1 or WOX6 were identified. Thus, *AthWOX2*‐like genes underwent recent gene duplication with potential loss that was not detected for other T3 WOXes.

### Dynamic genomic processes contributed to gene family evolution

**Figure 7:**
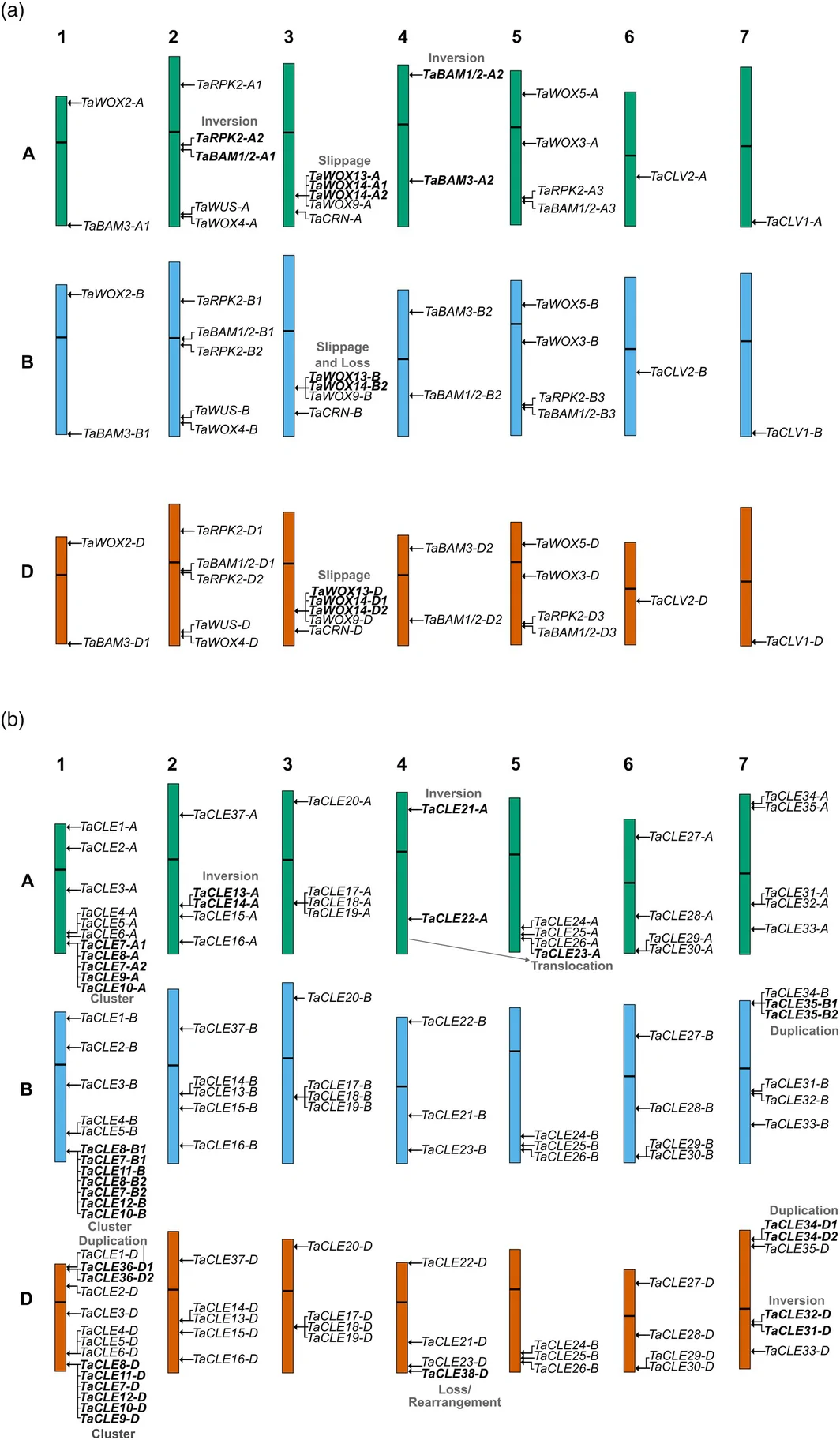
Chromosomal locations of *Triticum aestivum* homoeologues of CLAVATA pathway genes. (a) The location of *TaCLV1*, *TaBAM*, *TaCLV2*, *TaCRN*, *TaRPK2* and T3 *TaWOX* genes on the *T. aestivum* genome, as determined using Ensembl Plants (Harrison et al., 2023). The positions of inversions and gene losses are highlighted in bold text. (b) The location of *TaCLE* genes as determined using Ensembl Plants (Harrison et al., 2023), highlighting the position of duplications, inversions, rearrangement, gene clustering, loss and translocation. Chromosome numbers are shown at the top and the subgenome of each chromosome is indicated to the left.

This study identified multiple *T. aestivum* homoeologues of *CLV1*, *BAM1/2*, *BAM3*, *CLV2*, *CRN*, *RPK2*, *CLV3* and multiple T3 *WOX*es (Figures 2, 3, 4, 5, 6). To help us understand the evolutionary processes acting on these gene families in wheat, the chromosomal locations of these genes were mapped against the IWGSC genome assembly (International Wheat Genome Consortium (IWGSC), 2018) (Figure 7a). All genes comprising homoeologous triads were located on homologous chromosomes, and most gene triads were syntenic, with conserved positions of each homoeologue on the A, B and D chromosomes (e.g. *TaWOX2* and *TaBAM3‐1‐A* on chromosome 1). However, the positions of *TaRPK2‐2* and *TaBAM1/2‐1* on chromosome 2 and *TaBAM1/2‐2* and *TaBAM3‐2* on chromosome 4 were inverted on the A genome with respect to the B and D genomes (Figure 7a), and the latter inversion is consistent with previous reports of a pericentric inversion of the short arm and long arm of chromosome 4A (Dvorak et al., 2018). Mapping chromosome positions also revealed *TaWOX13 and TaWOX14* gene clustering on chromosome 3 (Figure 7a). As these genes formed sister clades with A, B and D genome representatives in our phylogenetic analysis, we propose that *TaWOX13* and *TaWOX14* arose from a duplication in the ancestor of the wheat species included in our analysis. The proximity of *TaWOX14‐1* and *TaWOX14‐2* on A and D chromosomes and extra TaWOX14‐D1, TaWOX14‐A1 and TduWOX14‐A1 sequences at the base of the WOX13 clade in our phylogenetic analysis suggest more recent tandem gene duplications, perhaps by slippage or recombination errors. The lack of *TaWOX14‐2* on chromosome 4B also suggests potential gene loss (Figure 7a).

The position of all 112 *T. aestivum CLE* homoeologues was mapped separately for ease of visualisation, again showing that most homoeologous genes were syntenic (Figure 7b). However, the positions of *TaCLE21* and *TaCLE22* were inverted on chromosome 4A with respect to their positions on chromosomes 4B and 4D, a previously unreported translocation. While *TaCLE23‐B* and *TaCLE23‐D* were located on chromosomes 4B and 4D, *TaCLE23‐A* was located on chromosome 5A, consistent with a previously identified partial translocation of the long arms of chromosome 4A and 5A in a progenitor of *T. aestivum* (Dubcovsky et al., 1996). The absence of *TaCLE38* but retention of neighbouring *TaCLE23* and *TaCLE21* genes suggests a gene loss from the A and B genomes. On chromosome 7, there were independent duplications of *TaCLE34* and *TaCLE35* on the B and D genomes, and an inversion of *TaCLE32* and *TaCLE31* positions on the D genome with respect to the A and B genomes. There was a similar inversion of *TaCLE13* and *TaCLE14* positions on chromosome 2A with respect to chromosome 2B and 2D (Figure 7b). There were more *TaCLE*s on chromosome 1 than on the other chromosomes, and although the positions of *TaCLE1*, *TaCLE2*, *TaCLE3*, *TaCLE4*, *TaCLE5* were conserved, the positions of the remaining *TaCLE*s were not, with a loss of *TaCLE6* on chromosome B, and gains of *TaCLE36‐D1* and *TaCLE36‐D2* on chromosome D. There was a *TaCLE* cluster of five to seven genes on the long arm of chromosome 1 with *TaCLEs 7‐10* occupying different positions relative to other *TaCLE*s on each chromosome, in a pattern that is consistent with independent duplications, deletions and inversions.

By comparing gene copy numbers in diploid species, and rice, maize and Arabidopsis, we were able to estimate baseline copy numbers in polyploid wheat (Table 1). While *CLV1* and *CRN* had a baseline copy number of 1 in all species, *CLV2* could not be identified in *T. urartu* but otherwise had a single baseline copy. In accordance with Vardanega et al. (2025), the baseline quotient of *BAM*s was 5 in grasses, with *T. turgidum* ssp. *durum* gaining an extra copy on chromosome 1. *RPK2* had baseline copy numbers of 3 in wheat, with the loss of a *T. urartu* copy. The baseline copy numbers of *CLE*s and T3 *WOX*es were more variable, in line with results from our phylogenetic and synteny analyses.

**Table 1:** The number of *CLV1*, *BAM1/2*, *BAM3*, *CLV2*, *CRN*, *RPK2*, *CLE* and T3 *WOX*es in Triticeae species, rice maize and Arabidopsis

Species	Ploidy level	*CLV1* homologues	*BAM* homologues	*CLV2* homologues	*CRN* homologues	*RPK2* homologues	T3 *WOX* homologues	*CLE* homologues
*Triticum aestivum*	Hexaploid	1 (3)	5 (15)	1 (3)	1 (3)	3 (9)	9 (26)	31 (112)
*Triticum turgidum* subsp. *durum*	Tetraploid	1 (2)	5 (11)	1 (2)	1 (2)	3 (6)	9 (17)	27 (50)
*T. turgidum* subsp. *dicoccoides*	Tetraploid	1 (2)	5 (10)	1 (2)	1 (2)	3 (6)	8 (15)	32 (53)
*Triticum urartu*	Diploid	1	5	0	1	2	6	30
*Aegilops tauschii*	Diploid	1	5	1	1	3	9	36
*Oryza sativa Japonica*	Diploid	1	5	1	1	2	6	47
*Zea mays*	Diploid	1	7	1	1	3	10	41
*Arabidopsis thaliana*	Diploid	1	3	1	1	2	8	31

Inferred baseline copy numbers are shown to the left, and bracketed numbers show the number of homoeologous genes identified in *T. aestivum*, *T. turgidum* ssp. *durum* and *T. turgidum* ssp. *dicoccoides*.

### The expression of genes encoding receptor‐like kinases and TaWOXes was mostly balanced

**Figure 8:**
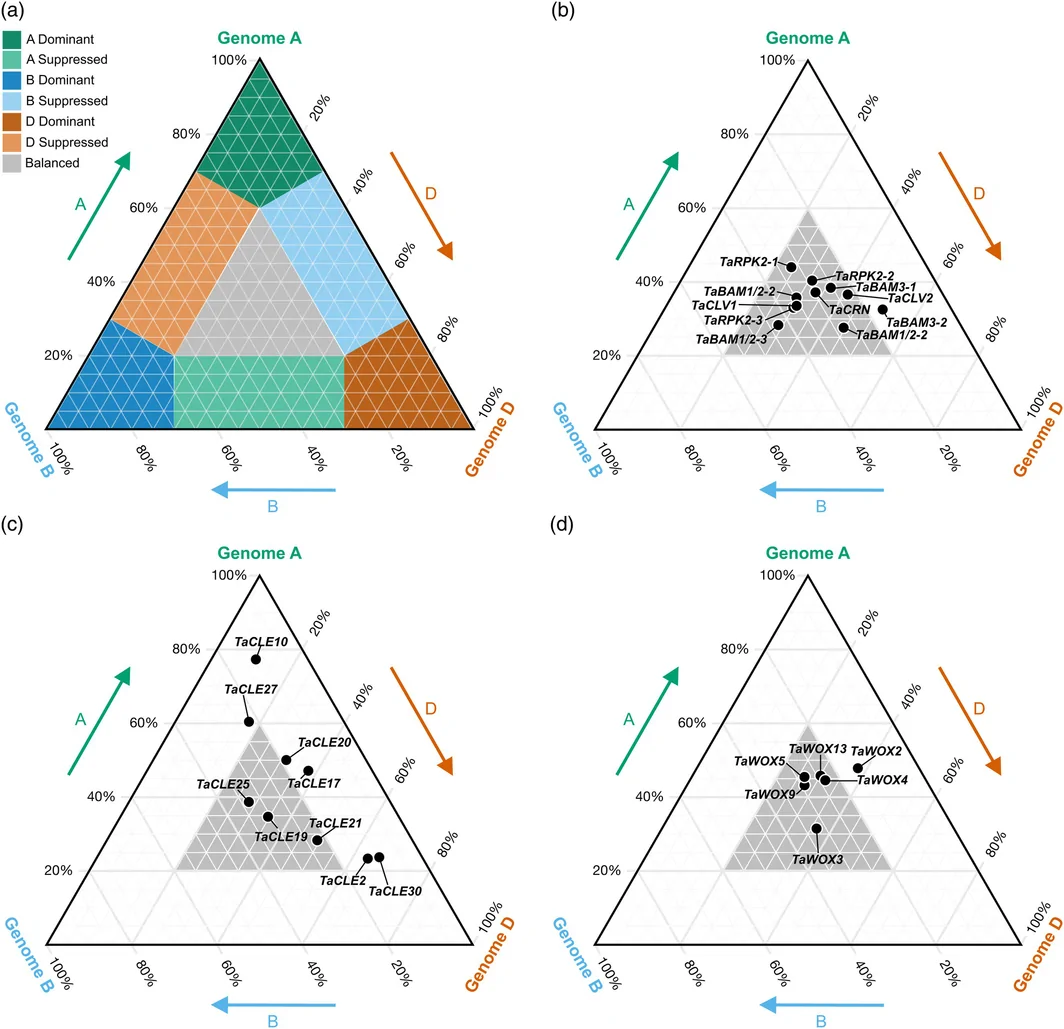
Relative expression levels of receptor‐like kinase, *TaCLE* and *TaWOX* homoeologues. (a) A ternary plot with axes representing the expression level of respective homoeologues from the A, B and D genomes. Gene triads located in dark green, dark blue or dark orange segments respectively show stronger expression from the A, B or D subgenome, while triads located in light green, light blue and light orange segments show suppression of A, B or D homoeologue expression. Triads located in the central grey triangle show balanced expression. (b) Receptor‐like kinase gene triads mostly showed balanced expression, but the B genome copy of *TaBAM3‐2* was suppressed. (c) The expression of *TaCLE* triads was mostly imbalanced between A, B and D genome copies. *TaCLE10* showed A dominant expression and *TaCLE2* and *TaCLE30* were D dominant. (d) *TaWOX* triads mostly showed balanced expression, but the B genome copy of *TaWOX2* was suppressed.

To explore the possibility for homoeologue non‐functionality, we retrieved homoeologous gene triad expression data in each gene family from the Wheat Expression Browser (Ramírez‐González et al., 2018; Winter et al., 2007). The data averaged expression from 90 Chinese Spring RNASeq samples from multiple tissues including leaves, roots, spikes and grain from plants grown under non‐stressed conditions (Ramírez‐González et al., 2018, Winter et al., 2007). The relative expression of homoeologues from each genome was visualised using ternary plots, where the axes represent % expression from each genome and imbalances in expression can be clearly visualised (Figure 7a). These analyses showed that except for *TaBAM3‐2* which had B genome suppression, all the receptor‐like kinase encoding genes had balanced expression (Figure 7b). Amongst the *WOX* genes, only *TaWOX2* showed imbalanced expression, with B genome suppression. However, amongst the 12 CLE triads identified in Figure 5(b), only *TaCLE19*, *TaCLE21* and *TaCLE25* had balanced expression. The A homoeologue of *TaCLE10* showed dominant expression, and D homoeologues of *TaCLE2* and *TaCLE30* were dominantly expressed. *TaCLE17* and *TaCLE20* showed B suppression and *TaCLE27* was D suppressed. No data for *TaCLE28*, *TaCLE33* and *TaCLE37* was retrieved as these genes were not annotated in the genome (International Wheat Genome Consortium (IWGSC), 2018). Overall, the expression analyses demonstrated the retention of functional A, B and D genome copies of wheat receptor‐like kinases and WOXes and indicated that *TaCLE* homoeologues might have more specialist roles than other genes as their expression was less balanced (Figure 8).

## DISCUSSION

### There may be stabilising selection on receptor‐like kinases and WOXes

To summarise, the data presented identified CLAVATA pathway genes in wheat and the wider Triticeae, providing insight into the evolution of gene families within the Triticeae and identifying *T. aestivum* candidates for future functional work. At present, there are limited expression data (Long et al., 2024; Ramírez‐González et al., 2018; Winter et al., 2007) and little functional work (Li et al., 2019) on these genes, so we are unable to compare spatiotemporal patterns of expression in relation to the evolution of grass inflorescence architecture or other traits. However, intercepting wheat CLAVATA pathway function has been identified as a priority for efforts to engineer crop yield improvements (Fletcher, 2018), and gene functions will likely be revealed in the next 5–10 years. Baseline numbers of receptor‐like kinase genes in our study were mostly consistent with previously reported numbers from rice and maize. Maize (*ZmTD1*), rice (*OsFON1*) and barley (*HvCLV1*) *CLV1* orthologues (Suzaki et al., 2004; Vardanega et al., 2025), maize (*ZmFEA2*) and rice (*OsCLV2*) *CLV2* orthologues and maize (*ZmCRN*) and rice (*OsCRN*) CRN orthologues have been functionally characterised (Je et al., 2018; Li et al., 2024). Combined with our data, published data suggest that the last common ancestor of grasses had a single *CLV1*, *CLV2* and *CRN* gene. The number of Triticeae *BAM*s corresponded to the number of rice and barley but not maize *BAM*s (Vardanega et al., 2025), and a sister relationship between *ZmBAM3‐3* and *ZmBAM1/2‐4* and other maize genes suggests that *BAM* duplications occurred in maize following its divergence from an ancestor shared with wheat and rice (Gaut, 2001). In contrast, shared baseline *RPK2* copy numbers of 3 in maize and most Triticeae but not rice suggest that rice underwent post‐speciation gene loss. The expression of *TaCLV1*, *TaBAM*, *TaCRN*, *TaCLV2* and *TaRPK2* genes was almost exclusively balanced, indicating potential stabilising selection on receptor‐like kinase function. *TaWOX13/TaWOX14* copy number variability in the AthWOX2 clade but not other T3 WOX clades, and the chromosomal proximity of paralogues, suggests recent duplications of these genes, with stabilising selection on genes in the Triticeae WOX13 and WOX14 clades and remaining sequences undergoing drift. *TaCLE*s had greater copy number and structural variability than other genes, with some genes encoding multiple CLE domains and many recent chromosomal rearrangements. A minority of *TaCLE*s showed balanced expression and hence we suggest that *TaCLE*s are unlikely to be under stabilising selection as for other genes included in our study.

### Distinct selection pressures on *T. aestivum* Type A and Type B TaCLEs

In line with previously published work (Goad et al., 2017, Li et al., 2019), TaCLEs lacked phylogenetic signal. With low support, a minority of genes formed triad clades, and distinct A and B‐type clades were also resolved (Figure 5). Of 112 *TaCLE*s homoeologues identified, eight were newly identified (Li et al., 2019), perhaps due to a tendency for *CLEs* to be overlooked in genome annotation pipelines (Hastwell et al., 2015). Consistently, many *TaCLE*s were not annotated in respective genome assemblies, and we were unable to retrieve expression patterns for these genes. High Type A CLE copy number variability between taxa in our sample suggests multiple gene losses and gains, and this finding is consistent with broader phylogenetic comparisons within land plants and the high evolvability of CLE expression patterns and functions (Jun et al., 2010; Nemec‐Venza et al., 2025; Whitewoods et al., 2018). Notably, however TDIF‐like CLEs were present as a single copy per genome, suggesting that distinct selection pressures may act on Type A and Type B *TaCLE*s, as well as *TaCLE*s and the other gene families we sampled. *CLE* expression analyses in other species have shown that expression is frequently in tightly restricted domains (Jun et al., 2010; Nemec‐Venza et al., 2025), and that selection is likely to act on the promoter regions to generate new signalling sites (Hobe et al., 2003), and this may also be the case in wheat.

### Inconsistent sister relationships between *T. aestivum* subgenome clades

Large‐scale phylogenetic analysis has resolved that *T. aestivum* A and B subgenomes are more closely related to the D genome than to each other (Marcussen et al., 2014). In all the maximum likelihood trees generated here, clades containing subgenome sequences from different taxa consistently had Triticeae sister clades from other subgenomes, and for receptor‐like kinases and T3 *TaWOX* genes, the A and D subgenome clades were usually more closely related than other subgenome clades, perhaps due to drift. 26% of Triticeae clades had A and B genes as sisters to each other and jointly sisters to D, 47% had A and D jointly sisters to B and 26% had B and D as jointly sisters to A. All 12 *TaCLE* triad clades had A and B genome sisters, with a joint D sister, but relationships between genomes in the rest of the CLE tree were unclear. Following polyploidisation, genome conflicts can lead to biased retention of one parental genome and orthologue loss (Emery et al., 2018), yet signal transduction genes may be preferentially retained due to selection to maintain dosage balance amongst interacting gene products (Blanc & Wolfe, 2004). Previous work in other species has shown that the CLAVATA pathway is genetically buffered (Rodriguez‐Leal et al., 2019), such that loss of function of one component is compensated by changes in the function of another component; for instance in Arabidopsis, *BAM* expression is up‐regulated and broadened to compensate for loss of *CLV1* function (Nimchuk et al., 2015). Retention of all three orthologues could indicate that the balance between subgenome copies is important in *T. aestivum* CLAVATA function. Our data provide a platform to explore these ideas in future analyses of gene function.

## METHODS

### Sequence retrieval and validation

*Triticum aestivum*, *T. turgidum* ssp. *durum*, *T. turgidum* ssp. *dicoccoides*, *T. urartu* and *A. tauschii* were sampled to illuminate subgenome evolution patterns in Triticeae, and *O. sativa* and *Z. mays* were sampled as non‐Triticeae monocot outgroups (Harrison et al., 2023). Using Ensembl Plants (Harrison et al., 2023), Arabidopsis CLAVATA pathway peptide sequences were retrieved and used as queries in tBLASTn searches with a threshold value of e^−1000^ for *CLV1*, *BAM1*, *BAM2*, *BAM3*, *CLV2*, *CRN* and *RPK2* and e^−100^ for *WOX* and *CLE* queries. Initial hits were validated by reciprocal BLASTs against the Arabidopsis genome using Ensembl Plants (Harrison et al., 2023), and retained if the top hit was the original Arabidopsis gene of interest. The Ensembl Plants Compara tool (Harrison et al., 2023) and OrthoFinder2 (Emms & Kelly, 2019) were also used to search genomes for genes within the same orthogroup as Arabidopsis genes of interest, with OrthoFinder2 searches using the strategy outlined by Harris et al. (2020). Searches that retrieved Arabidopsis genes of interest from two out of three of these methods were taken forward for multiple sequence alignment and tree generation.

### Multiple sequence alignment and phylogenetic tree generation

To align *CLV1/BAM*, *CLV2*, *CRN*, *RPK2* and T3 *WOX* sequences, coding DNA sequences were translated into amino acid sequences and aligned using MUSCLE (Edgar, 2004) in MEGA11 (Tamura et al., 2021). Full‐length alignments were then manually edited using UniproUGENE (Okonechnikov et al., 2012) to remove positions with a number of gaps above a specified threshold. After trialling different thresholds, thresholds were set at 10% for CLV1/BAM and WOX sequences, as this balanced the strong alignment of conserved regions with the requirement for phylogenetically informative characters from regions with higher sequence variability. 10% thresholds were too sensitive for CLV2 and CRN, resulting in the removal of positions with a single gap, so thresholds were raised to 20%. The threshold was raised to 35% for RPK sequences to enable us to include RPK1 and RPK2‐1, which lack large parts of the LRR domain, in the phylogenetic analysis. The WOX alignment was edited to ensure correct alignment of the conserved WUS‐box motif (van der Graaff et al., 2009). These final alignments were then used to generate maximum likelihood trees using the JTT model of sequence evolution (Jones et al., 1992) in MEGA11 (Tamura et al., 2021). CLE sequences outside of the conserved CLE domain are highly divergent (Goad et al., 2017; Whitewoods et al., 2018), and therefore the CLE domain alone was taken forward for analysis, and a neighbour‐joining strategy was taken using TreeViewer (Bianchini & Sánchez‐Baracaldo, 2024). For all trees, 1000 bootstrap replicates were performed and support values of over 50% are shown on branches; values of over 80% can be considered significant. CLE domain consensus sequences were generated and plotted using UniproUGENE (Okonechnikov et al., 2012).

### Gene nomenclature

Previously identified Triticeae genes were named following precedent (Li et al., 2019, 2020; Shi et al., 2021) and newly identified genes were named in relation to Arabidopsis homologues and following nomenclatural guidelines (Boden et al., 2023). Rice and maize genes were named following database precedent (Kawahara et al., 2013; Sakai et al., 2013; Woodhouse et al., 2021), or if no precedent was given, were named by their homology to Triticeae genes. Rice and maize CLEs that were not previously named were represented by their gene IDs due to a lack of meaningful phylogenetic signal.

### Synteny analysis

The location of *T. aestivum* genes was mapped using the Ensembl Plants whole genome location‐based display tool (Harrison et al., 2023).

### Expression analyses

Gene expression data were retrieved from the developmental gene expression atlas, Wheat Expression Browser (Ramírez‐González et al., 2018; Winter et al., 2007). Averaged expression across all tissues sampled (Table S9) was used to generate ternary plots using a ternary plot tool (https://www.ternaryplot.com/). Expression data from *TaCLE* genes that were unannotated could not be retrieved and were excluded from the analysis.

## AUTHOR CONTRIBUTIONS

All authors contributed to the study design. SJC developed the pipeline to identify orthologues, implemented OrthoFinder 2 searches and prepared preliminary figures and trees. KJ completed the searches, figures and trees. SJC, KJ and CJH drafted the manuscript and all authors commented on drafts. KJ and CJH revised the manuscript after review.

## CONFLICT OF INTEREST

The authors declare no conflicts of interest.

## Supporting information


**Table S1.** The species selected for sampling in tBLASTn searches and their respective genome assemblies and IDs. Five Triticeae species were selected, with rice (*O. sativa*), maize (*Z. mays*) and Arabidopsis included as outgroups.
**Table S2.** List of CLV1 and BAM‐like sequences included in Figure 2 with the species of origin, gene ID and genomic location of Triticeae genes. Triticeae genes were named according to their homology to Arabidopsis CLV1 or BAM genes, following wheat gene nomenclature guidelines (Boden et al., 2023).
**Table S3.** List of CLV2‐like sequences included in Figure 3 with the species of origin, gene ID and genomic location Triticeae genes were named for their homology to Arabidopsis CLV2, following wheat gene nomenclature guidelines (Boden et al., 2023).
**Table S4.** List of CRN‐like sequences included in Figure 3 with the species of origin, gene ID and genomic location of Triticeae genes. Triticeae genes were named for their homology to Arabidopsis CRN, following wheat gene nomenclature guidelines (Boden et al., 2023).
**Table S5.** List of RPK2‐like sequences included in Figure 4 with the species of origin, gene ID and genomic location of Triticeae genes. Triticeae genes were named following their homology to Arabidopsis RPK2, following wheat gene nomenclature guidelines (Boden et al., 2023).
**Table S6.** List of CLE‐like sequences included in Figure 5 with the species of origin, gene ID and genomic location of Triticeae genes. *T. aestivum* genes were named following precedent (Li et al., 2019) and newly identified *T. aestivum* genes were named following homoeology to *T. aestivum* genes, or following wheat nomenclature guidelines (Boden et al., 2023).
**Table S7.** List of Triticeae CLE‐like sequences not included in Figure 5 with the species of origin, gene ID and genomic location.
**Table S8.** List of T3 WOX‐like sequences included in Figure 6, with the species of origin, gene ID and genomic location of Triticeae genes. *T. aestivum* genes were named following precedent (Li et al., 2020 and Shi et al., 2021) and newly identified Triticeae genes were named in relation to the wheat genes.
**Table S9.** Averaged expression levels (transcripts per million) of *Triticum aestivum* CLAVATA pathway genes from the Wheat Expression Browser (Ramírez‐González et al., 2018; Winter et al., 2007). Expression data were averaged from Chinese Spring RNASeq data from different tissues under non‐stress conditions. *N* represents the number of samples in each tissue type.

## Data Availability

The data that supports the findings of this study are available in the Supplementary Material of this article.
