# Correction to “Phylogeny, chromosomal mapping and expression analyses of wheat CLAVATA pathway components suggest differential selection on receptor‐like kinases, CLEs and T3 WOXes”

**DOI:** 10.1111/tpj.71165

**Published:** 2026-10-07

Jeal, K. , Carpenter, S. J. , Burt, C. , Edwards, K. J. and Jill Harrison, C. (2025), Phylogeny, chromosomal mapping and expression analyses of wheat CLAVATA pathway components suggest differential selection on receptor‐like kinases, CLEs and T3 WOXes. The Plant Journal, 124, e70580. 10.1111/tpj.70580.PMC1267792041343720

In the Acknowledgements section, funding from BBSRC grant BB/T008741/1 was inadvertently omitted.

We apologize for this error and thank the BBSRC for funding this work.

The published article has been updated to reflect this correction.

